# Supplementary material for: Metabolic phenotyping by treatment modality in obese women with gestational diabetes suggests diverse pathophysiology: An exploratory study
Source: PLoS One. 2020 Apr 2;15(4):e0230658. doi: 10.1371/journal.pone.0230658 (PMC7117764; doi:10.1371/journal.pone.0230658)
Supplement: S1 Table — (DOCX) [file pone.0230658.s001.docx]

S1 Table:

| **Analyte** | **Sample** | **Method** | **Platform** | **Coefficient of Variation (%) ^*^** |
| --- | --- | --- | --- | --- |
| Insulin | Plasma | Electrochemiluminescence immunoassay | Roche, Cobas e411 | < 10.3 |
| C-peptide | Serum | Electrochemiluminescence immunoassay | Roche, Cobas e411 | < 6.2 |
| Fructosamine | Plasma | Colorimetric, nitroblue tetrazolium | Roche, Cobas c311 | < 3.4 |
| gGT | Plasma | Enzymatic, colorimetric | Roche, Cobas c311 | < 3.9 |
| SHBG | Serum | Electrochemiluminescence immunoassay | Roche, Cobas e411 | < 7.9 |
| Adiponectin | Plasma | Enzyme-linked immunosorbent assay | R and D Systems | < 6.9 |

*highest coefficient of variation from either time point 1 or 2 quoted. gGT γ-glutamyl transferase, SHBG sex hormone binding globulin
