# Supplementary material for: Metabolic phenotyping by treatment modality in obese women with gestational diabetes suggests diverse pathophysiology: An exploratory study
Source: PLoS One. 2020 Apr 2;15(4):e0230658. doi: 10.1371/journal.pone.0230658 (PMC7117764; doi:10.1371/journal.pone.0230658)
Supplement: S2 Table — (DOCX) [file pone.0230658.s002.docx]

S2 Table: Comparison of GDM women in treatment modality cohort compared to those excluded (control arm)

|  | **GDM treatment (*n*=71)** | **Excluded (*n*=103)** | ***P* value*** |
| --- | --- | --- | --- |
|  | **Mean (SD)/ Median (IQR)/ *n* (%)** | **Mean (SD)/ Median (IQR)/ *n* (%)** |  |
| Age (years) | 32.2 (5.2) | 31.1 (5.0) | 0.14 |
| BMI (kg/m^2^) | 36.3 (33-39.9) | 36.4 (33.8-40.4) | 0.52 |
| **Ethnicity** |  |  |  |
| African | 14 (19.7) | 18 (17.5) |  |
| African Caribbean | 6 (8.5) | 7 (6.8) |  |
| South Asian | 4 (5.6) | 8 (7.8) | 0.33 |
| European | 40 (56.3) | 67 (65.0) |  |
| Other | 7 (9.9) | 3 (2.9) |  |
| **Parity** |  |  |  |
| Nulliparous | 34 (47.9) | 42 (40.8) | 0.35 |
| **Centre** |  |  |  |
| St Thomas' Hospital | 39 (54.9) | 34 (33.0) |  |
| Kings College Hospital^ | 0 | 16 (15.5) |  |
| Newcastle | 8 (11.3) | 15 (14.6) |  |
| Glasgow | 16 (22.5) | 9 (8.7) | <0.01 |
| Manchester | 3 (4.2) | 7 (6.8) |  |
| Bradford | 1 (1.4) | 3 (2.9) |  |
| Sunderland^ | 0 | 9 (8.7) |  |
| St Georges Hospital | 4 (5.6) | 10 (9.7) |  |
| **Pregnancy outcome** |  |  |  |
| GA at delivery | 38.7 (38.1-39.7) | 38.7 (38-40.3) | 0.51 |
| Birthweight (g) | 3335.6 (448.9) | 3435.7 (605.7) | 0.24 |
| LGA (customised) | 7 (9.9) | 12 (11.9) | 0.68 |
| BW (customised centile) | 53.9 (26.8-77.5) | 58.6 (32.8-77) | 0.52 |

**p* value from χ^2^, Student’s *t* test or Mann–Whitney test as appropriate. GDM gestational diabetes, GA gestational age, LGA large by gestational age, BW birthweight, SD standard deviation, IQR interquartile range. ^No research blood samples were received from these centres.
