# Supplementary material for: Metabolic phenotyping by treatment modality in obese women with gestational diabetes suggests diverse pathophysiology: An exploratory study
Source: PLoS One. 2020 Apr 2;15(4):e0230658. doi: 10.1371/journal.pone.0230658 (PMC7117764; doi:10.1371/journal.pone.0230658)
Supplement: S3 Table — (DOCX) [file pone.0230658.s003.docx]

S3 Table: Absolute analyte concentrations by treatment modality, time point 1, 10 weeks before diagnosis/treatment (mean 17^+0^ weeks’)

|  | **No GDM** | **GDM** | | |
| --- | --- | --- | --- | --- |
| **Analyte, absolute units, time point 1** | **(*n=*229)** | **Diet (*n=*28)** | **Metformin (*n=*20)** | **Insulin (*n=*23)** |
|  | mean (SD)/median (IQR) | mean (SD)/ median (IQR) | mean (SD)/ median (IQR) | mean (SD)/ median (IQR) |
| Total lipids in chylomicrons and extremely large VLDL (umol/l) | 13.2 (6.6 - 22.6) | 16.1 (8.2 - 26.3) | 17.7 (10.3 - 23) | 17.9 (10.5 - 24.4) |
| Total lipids in very large VLDL (umol/l) | 45.9 (27.7 - 74.1) | 50.8 (25.3 - 83.4) | 58.8 (35.7- 76.2) | 65.6 (44.1 - 76.5) |
| Total lipids in large VLDL (umol/l) | 206 (139 - 206) | 223 (124 - 223) | 235 (166 - 235) | 275 (194 - 275) |
| Total lipids in medium VLDL (mmol/l) | 0.5 (0.19) | 0.48 (0.2) | 0.54 (0.23) | 0.53 (0.16) |
| Total lipids in small VLDL (mmol/l) | 0.57 (0.15) | 0.54 (0.14) | 0.58 (0.13) | 0.62 (0.14) |
| Total lipids in very large HDL (mmol/l) | 0.72 (0.18) | 0.82 (0.24) | 0.72 (0.19) | 0.69 (0.21) |
| Total lipids in large HDL (mmol/l) | 1.17 (0.26) | 1.32 (0.33) | 1.21 (0.24) | 1.23 (0.34) |
| Total cholesterol in VLDL (mmol/l) | 0.7 (0.17) | 0.67 (0.15) | 0.69 (0.16) | 0.75 (0.2) |
| Total cholesterol in HDL (mmol/l) | 1.84 (0.25) | 1.96 (0.33) | 1.88 (0.21) | 1.96 (0.36) |
| Serum total triglycerides (mmol/l) | 1.24 (0.36) | 1.24 (0.4) | 1.33 (0.43) | 1.34 (0.31) |
| Triglycerides in VLDL (mmol/l) | 0.75 (0.29) | 0.73 (0.32) | 0.84 (0.37) | 0.81 (0.23) |
| Triglycerides in HDL (mmol/l) | 0.16 (0.03) | 0.18 (0.03) | 0.17 (0.03) | 0.18 (0.04) |
| Mean diameter for VLDL particles (nm) | 36.3 (0.93) | 36.4 (1.17) | 36.8 (1.1) | 36.5 (0.86) |
| Mean diameter for LDL particles (nm) | 23.6 (0.06) | 23.6 (0.06) | 23.6 (0.05) | 23.6 (0.04) |
| Mean diameter for HDL particles (nm) | 10.3 (0.18) | 10.3 (0.21) | 10.3 (0.18) | 10.2 (0.19) |
| Ratio of polyunsaturated fatty acids to total fatty acids (%) | 35.4 (2.37) | 35.6 (2.91) | 34.6 (2.6) | 35.3 (2.36) |
| Ratio of monounsaturated fatty acids to total fatty acids (%) | 27.5 (1.86) | 27.5 (2.22) | 27.8 (1.7) | 27.8 (1.54) |
| Ratio of saturated fatty acids to total fatty acids (%) | 37.1 (1.26) | 36.9 (1.36) | 37.6 (1.23) | 37 (1.27) |
| Isoleucine (umol/l) | 44.8 (12.3) | 49.6 (12.2) | 49.5 (14.9) | 45.6 (8.84) |
| Alanine (umol/l) | 376 (38.1) | 388 (42.7) | 386 (35.4) | 376 (39.8) |
| Glucose (mmol/l) | 3.69 (0.51) | 4.05 (0.77) | 4.13 (1) | 4.14 (0.59) |
| Insulin (mU/l) | 21.4 (11.6 - 44.4) | 33.6 (19.4 - 79.9) | 37.7 (25 - 114.6) | 26.5 (13.8 - 52.9) |

GDM gestational diabetes, VLDL very large density lipoprotein, HDL high density lipoprotein, LDL low density lipoprotein. SD standard deviation, IQR interquartile range
