# Supplementary material for: Metabolic phenotyping by treatment modality in obese women with gestational diabetes suggests diverse pathophysiology: An exploratory study
Source: PLoS One. 2020 Apr 2;15(4):e0230658. doi: 10.1371/journal.pone.0230658 (PMC7117764; doi:10.1371/journal.pone.0230658)
Supplement: S4 Table — (DOCX) [file pone.0230658.s004.docx]

S4 Table: Absolute analyte concentrations by treatment modality, time point 2, at time of OGTT (mean 27^+5^ weeks’)

|  | **No GDM** | **GDM** | | |
| --- | --- | --- | --- | --- |
| **Analyte, absolute units, time point 2** | **(*n*=229)** | **Diet (*n=*28)** | **metformin (*n=*20)** | **Insulin (*n=*23)** |
|  | **mean (SD)/median (IQR)** | **mean (SD)/ median (IQR)** | **mean (SD)/ median (IQR)** | **mean (SD)/ median (IQR)** |
| Total lipids in chylomicrons and extremely large VLDL (umol/l) | 20.5 (12 - 31.5) | 15.3 (10.3 - 24.8) | 27.9 (13.7 - 47.2) | 32.9 (19.7 - 47.8) |
| Total lipids in very large VLDL (umol/l) | 72.6 (47.1 - 105) | 59.1 (42.2 - 81.8) | 90 (59.9 - 153.5) | 113 (63.9 - 113) |
| Total lipids in large VLDL (umol/l) | 306 (221 - 306) | 257 (192 - 257) | 339 (258 - 339) | 414 (275 - 414) |
| Total lipids in medium VLDL (mmol/l) | 0.67 (0.25) | 0.56 (0.24) | 0.75 (0.34) | 0.78 (0.27) |
| Total lipids in small VLDL (mmol/l) | 0.72 (0.19) | 0.61 (0.17) | 0.73 (0.21) | 0.76 (0.2) |
| Total lipids in very large HDL (mmol/l) | 0.8 (0.2) | 0.9 (0.24) | 0.81 (0.24) | 0.71 (0.24) |
| Total lipids in large HDL (mmol/l) | 1.24 (0.28) | 1.39 (0.33) | 1.27 (0.32) | 1.18 (0.33) |
| Total cholesterol in VLDL (mmol/l) | 0.88 (0.23) | 0.77 (0.19) | 0.88 (0.24) | 0.9 (0.25) |
| Total cholesterol in HDL (mmol/l) | 1.89 (0.28) | 2.01 (0.34) | 1.9 (0.29) | 1.87 (0.31) |
| Serum total triglycerides (mmol/l) | 1.63 (0.49) | 1.45 (0.52) | 1.81 (0.64) | 1.82 (0.54) |
| Triglycerides in VLDL (mmol/l) | 1.01 (0.39) | 0.86 (0.4) | 1.17 (0.55) | 1.2 (0.42) |
| Triglycerides in HDL (mmol/l) | 0.2 (0.04) | 0.2 (0.04) | 0.21 (0.04) | 0.21 (0.05) |
| Mean diameter for VLDL particles (nm) | 36.7 (0.93) | 36.5 (1.14) | 37.2 (1.2) | 37.4 (1) |
| Mean diameter for LDL particles (nm) | 23.6 (0.05) | 23.6 (0.06) | 23.6 (0.06) | 23.6 (0.08) |
| Mean diameter for HDL particles (nm) | 10.3 (0.18) | 10.4 (0.19) | 10.3 (0.22) | 10.2 (0.21) |
| Ratio of polyunsaturated fatty acids to total fatty acids (%) | 33.9 (2.14) | 34.1 (2.31) | 32.3 (2.63) | 32.4 (2.3) |
| Ratio of monounsaturated fatty acids to total fatty acids (%) | 29.6 (1.49) | 29.6 (1.54) | 30.4 (1.49) | 30.5 (1.47) |
| Ratio of saturated fatty acids to total fatty acids (%) | 36.5 (1.14) | 36.3 (1.32) | 37.3 (1.41) | 37.1 (1.11) |
| Isoleucine (umol/l) | 42.9 (9.28) | 46.9 (8.84) | 50.4 (9.26) | 46.5 (9.93) |
| Alanine (umol/l) | 375 (37.3) | 378 (34.2) | 384 (38.1) | 388 (32.1) |
| Glucose (mmol/l) | 3.83 (0.85) | 3.86 (0.47) | 4.28 (0.69) | 4.66 (1.37) |
| Insulin (mU/l) | 18.1 (12.5 - 35.8) | 19.8 (13.4 - 28.6) | 22.1 (20.2 - 43.3) | 36.6 (18.3 - 51.4) |

GDM gestational diabetes, VLDL very large density lipoprotein, HDL high density lipoprotein, LDL low density lipoprotein. SD standard deviation, IQR interquartile range
