# Supplementary material for: Metabolic phenotyping by treatment modality in obese women with gestational diabetes suggests diverse pathophysiology: An exploratory study
Source: PLoS One. 2020 Apr 2;15(4):e0230658. doi: 10.1371/journal.pone.0230658 (PMC7117764; doi:10.1371/journal.pone.0230658)
Supplement: S5 Table — (DOCX) [file pone.0230658.s005.docx]

S5 Table: Absolute analyte concentrations by treatment modality, time point 3, following treatment (mean 34^+6^ weeks’)

|  | **No GDM** | **GDM** | | |
| --- | --- | --- | --- | --- |
| **Analyte, absolute units, time point 3** | **(*n=*229)** | **Diet (*n=*28)** | **Metformin (*n=*20)** | **Insulin (*n=*23)** |
|  | **mean (SD)** | **mean (SD)** | **mean (SD)** | **mean (SD)** |
| Total lipids in chylomicrons and extremely large VLDL (umol/l) | 33.9 (19.1 - 53.2) | 23.2 (13.3 - 36.7) | 32.7 (15.5 - 75.2) | 46.3 (23.9 - 65.6) |
| Total lipids in very large VLDL (umol/l) | 113 (75.8 - 113) | 88.3 (59.1 - 131.6) | 113 (68.8 - 113) | 151 (97.1 - 151) |
| Total lipids in large VLDL (umol/l) | 450 (325 - 450) | 320 (259 - 320) | 469 (332 - 469) | 622 (421 - 622) |
| Total lipids in medium VLDL (mmol/l) | 0.93 (0.38) | 0.79 (0.35) | 1.07 (0.51) | 1.1 (0.39) |
| Total lipids in small VLDL (mmol/l) | 0.92 (0.28) | 0.81 (0.24) | 0.99 (0.33) | 1.05 (0.27) |
| Total lipids in very large HDL (mmol/l) | 0.78 (0.2) | 0.86 (0.27) | 0.73 (0.24) | 0.77 (0.23) |
| Total lipids in large HDL (mmol/l) | 1.16 (0.31) | 1.31 (0.4) | 1.13 (0.32) | 1.19 (0.33) |
| Total cholesterol in VLDL (mmol/l) | 1.1 (0.32) | 0.98 (0.24) | 1.11 (0.34) | 1.24 (0.32) |
| Total cholesterol in HDL (mmol/l) | 1.8 (0.33) | 1.9 (0.42) | 1.73 (0.31) | 1.84 (0.33) |
| Serum total triglycerides (mmol/l) | 2.18 (0.73) | 1.95 (0.7) | 2.48 (0.96) | 2.54 (0.74) |
| Triglycerides in VLDL (mmol/l) | 1.43 (0.59) | 1.22 (0.57) | 1.68 (0.83) | 1.7 (0.61) |
| Triglycerides in HDL (mmol/l) | 0.23 (0.05) | 0.23 (0.05) | 0.24 (0.05) | 0.26 (0.05) |
| Mean diameter for VLDL particles (nm) | 37.2 (1.04) | 36.9 (1.14) | 37.5 (1.45) | 37.5 (1.08) |
| Mean diameter for LDL particles (nm) | 23.6 (0.05) | 23.6 (0.06) | 23.6 (0.11) | 23.6 (0.06) |
| Mean diameter for HDL particles (nm) | 10.3 (0.19) | 10.4 (0.23) | 10.2 (0.23) | 10.2 (0.2) |
| Ratio of polyunsaturated fatty acids to total fatty acids (%) | 33.2 (2.97) | 34.7 (3.45) | 32.6 (3.7) | 33.2 (2.78) |
| Ratio of monounsaturated fatty acids to total fatty acids (%) | 29.3 (2.11) | 28.3 (2.37) | 29.9 (2.42) | 29.8 (2.25) |
| Ratio of saturated fatty acids to total fatty acids (%) | 37.5 (1.36) | 37.1 (1.4) | 37.6 (2.18) | 37 (1.33) |
| Isoleucine (umol/l) | 48.9 (13) | 48.8 (14.9) | 52.6 (14.9) | 56.6 (14.4) |
| Alanine (umol/l) | 410 (44.4) | 394 (42.1) | 430 (42.9) | 430 (50.4) |
| Glucose (mmol/l) | 4.06 (0.78) | 4.44 (1.1) | 4.42 (1.04) | 4.56 (0.91) |
| Insulin (mU/l) | 46.9 (19.7 - 84) | 83.1 (29.7 - 150.1) | 55.7 (16.9 - 112.9) | 50.2 (18.7 - 157.8) |

GDM gestational diabetes, VLDL very large density lipoprotein, HDL high density lipoprotein, LDL low density lipoprotein. SD standard deviation, IQR interquartile range
