# Supplementary material for: Metabolic phenotyping by treatment modality in obese women with gestational diabetes suggests diverse pathophysiology: An exploratory study
Source: PLoS One. 2020 Apr 2;15(4):e0230658. doi: 10.1371/journal.pone.0230658 (PMC7117764; doi:10.1371/journal.pone.0230658)
Supplement: S1 Fig — (DOCX) [file pone.0230658.s006.docx]

S1 Fig:

Flow diagram: women with documented GDM treatment modality and complete biochemical data at trial time points 1 (mean 17^+0^ weeks’), 2 (mean 27^+5^ weeks’) and 3 (mean 34^+6^ weeks’) included in analyses of metabolite phenotypes by treatment modality

**71 GDM positive (IADPSG criteria)**

**229 GDM negative (IADPSG criteria)**

**28 on diet at term**

**23 on insulin at term**

**20 on metformin at term**

**300 women with complete biochemical data**

**338 excluded as incomplete biochemical data at time points 1, 2 and/or 3**

143 individuals did not give blood samples

**1303 women from UPBEAT RCT with OGTT results**

**3 excluded as treated GDM via local guidelines but IADPSG negative**

**23 excluded as positive via IADPSG but not GDM by local guidelines therefore not treated**

**639 women in intervention arm of RCT excluded**

**664 women in control arm of RCT**
